# Supplementary material for: Continuous extracorporeal femoral perfusion model for intravascular ultrasound, computed tomography and digital subtraction angiography
Source: PLoS One. 2023 May 23;18(5):e0285810. doi: 10.1371/journal.pone.0285810 (PMC10204951; doi:10.1371/journal.pone.0285810)
Supplement: S1 File — (PDF) [file pone.0285810.s001.pdf]

Examination Date	Start	End	t [min]	surgeons	t [min] per leg per surgeon	t [min] statistics
15.08.2022	08:05	10:15	130	2	130	130; 130
24.09.2022	09:28	11:28	120	1	60	60; 60
25.09.2022	09:20	10:41	81	1	40,5	40; 41
22.10.2022	invalid (additional cranial perfusion; preparation time not measurabe)					
23.10.2022	09:33	11:26	113	1	56,5	56; 57

without CT	Mean ± SD	130 ± 0
with CT	Mean ± SD	52 ± 9
Mann-Whitney-U (unilateral)		p=0,036

non contrast CT?
NO
YES
YES
N/A
YES
